# The Financial Aspect of the Probationer

**Published:** 1907-06-01

**Authors:** 


### Nursing Administration.

### THE FINANCIAL ASPECT OF THE PROBATIONER.

Theoretically the number of first-year probationers is a quarter of the entire staff. Practically the number varied in 1905 from 58.5 per cent. at St. George's Hospital and 60.3 at the Meath Hospital, Dublin (both including paying pupils) to 12.9 at the Charing Cross Hospital and 15.6 at the Dundee Royal Infirmary. The average in nine London hospitals with medical schools was 33.2; in six provincial hospitals with medical schools 27.1. In hospitals without medical schools the figures are:—Average of six London general hospitals, 29.1; average of 28 provincial general hospitals, 28.2. Among the nine hospitals with medical schools in London there are several which take a large number of paying pupils. If these were deducted the average for this group would be much the same as in the other hospitals—namely, just above the normal 25 per cent. required to renew the staff every three years. In order to get a fair idea of the financial cost of the probationer, it is necessary to consider, first, what expense her presence entails upon the hospital; and next, what the hospital receives from her in return.

The annual cost of the probationer is calculated with some exactitude in "Burdett's Annual." Taking it at the minimum it works out per head as follows:—

| | £ | s. |
|---|---|---|
| Collective expenses of the Nurses' Home, including rent, rates and taxes, water, light, heating, service, linen, cleaning, crockery etc., repairs, renewals, and miscellaneous items ... ... ... | 10 | 0 |
| Board ... ... ... ... ... ... ... ... | 20 | 0 |
| Laundry ... ... ... ... ... ... ... ... | 6 | 10 |
| Uniform ... ... ... ... ... ... ... ... | 1 | 10 |
| Lectures and examinations ... ... ... ... | 2 | 0 |
| Total (exclusive of salary) ... ... ... | 40 | 0 |

This is a very low estimate, the cost of board frequently amounting to £26 or £30 a year, and the collective expenses of the home being often higher than £10.

In addition to those things received by the probationer which have a direct monetary value, she is the recipient of a great deal of unpaid service in the way of instruction from all concerned with her, doctor, matron, ward sister, staff nurse. But it may fairly be contended on her side that the labour which she entails is more than compensated by the freshness she brings to her work.

It must now be considered what the hospital receives from the probationer. She is completely untrained in the work that she has to perform. She can bring no skill or knowledge, she has everything to learn. But she is no child. She is in the prime of life, at an age when, if she is ever to be of any use in the world, all her faculties are at their highest point of efficiency. She gives herself unreservedly. In other kinds of work there are opportunities for doing things for herself, seeing her friends, making her clothes, reading, or keeping up other pursuits, possibly supplementing her salary in sundry small ways. In hospital her entire time is surrendered to her duties with the exception of such recreation hours as are essential to health. She must be deaf to the call of family festivity, even, it may be, of family emergency. She must be content to be tired every day to the point of intense fatigue. She must devote every faculty and all her will to the end in view, the proper performance of duty. And she does not long remain unskilled. She is a probationer for three years, but it is only during the first six months that her labours are absolutely unskilled. By the middle of her second year she is a responsible person, able to take charge on occasion, and from then on she steadily grows in value to the institution. How do these things balance against what she receives from the institution? During her three probationary years she receives in kind, in board, lodging, and other benefits the equivalent of at least £120, and, in addition, entering the hospital an unskilled worker, she leaves it with a profession which will bring her a good living in almost any part of the world. There can hardly be two opinions about the fact that in her first year she is a debtor to the hospital, and is by no means able to render services equivalent to her maintenance. But does this right itself as her training goes on, so that at the end of her probation she may feel that she has not only repaid the benefits received, but is also entitled to a salary? Taking all things into consideration, we are disposed to think that the balance of benefit still remains on the side of the hospital, and that no injustice is done to the nurse when she serves her third year unpaid. Yet, with one or two exceptions, all hospitals pay their third-year probationers, and a small salary is commonly given also during the second year. The fact, however, that such salaries are paid does not completely establish the fact that the probationer is entitled to payment, on account of the value of her services. There are grave disadvantages in retaining in the service of the hospital a large body of unpaid workers, however liberally they may be recouped for their labours by comfortable maintenance and skilled instruction. And these drawbacks are so considerable that it is found in practice better by many institutions to pay a small salary, as pocket money, from the outset of the probationer's career. There are certain expenses in clothing, journeys, etc., which no nurse can escape, and if she is earning nothing she is apt to fall into debt, and so make a false start from the beginning. It is quite reasonable to expect the probationer to come provided with means to defray such little expenses, but the only way in which to ensure this is to require an entrance fee sufficient for the purpose, and return it as salary during the first year. This is the plan adopted by the Blackburn and East Lancashire Infirmary, the Nottingham General Hospital, the Swansea General Hospital, the Royal Hants County Hospital, the Adelaide Hospital (Dublin), and the Dublin House of Industry Hospitals. The plan has so much to recommend it that we are surprised it is not in more general use.

